# Multivalent Protein Assembly Using Monovalent Self-Assembling Building Blocks

**DOI:** 10.3390/ijms141021189

**Published:** 2013-10-22

**Authors:** Katja Petkau-Milroy, Michael H. Sonntag, Alexander Colditz, Luc Brunsveld

**Affiliations:** Laboratory of Chemical Biology and Institute of Complex Molecular Systems, Department of Biomedical Engineering, Eindhoven University of Technology, Den Dolech 2, Eindhoven 5612AZ, The Netherlands; E-Mails: k.petkau@tue.nl (K.P.-M.); m.sonntag@tue.nl (M.H.S.); a.colditz@student.tue.nl (A.C.)

**Keywords:** supramolecular polymers, protein-assembly, FRET, multivalency, biotin-streptavidin, self-assembling multivalency

## Abstract

Discotic molecules, which self-assemble in water into columnar supramolecular polymers, emerged as an alternative platform for the organization of proteins. Here, a monovalent discotic decorated with one single biotin was synthesized to study the self-assembling multivalency of this system in regard to streptavidin. Next to tetravalent streptavidin, monovalent streptavidin was used to study the protein assembly along the supramolecular polymer in detail without the interference of cross-linking. Upon self-assembly of the monovalent biotinylated discotics, multivalent proteins can be assembled along the supramolecular polymer. The concentration of discotics, which influences the length of the final polymers at the same time dictates the amount of assembled proteins.

## 1. Introduction

Protein assemblies regulate a plethora of biological processes. Signal transduction as well as immune-response depends on the spontaneous clustering of proteins in the membrane [1–3]. Self-assembled protein fibers such as actin and tubulin play an essential role in motility and stabilization of cells [4]. At the same time, mis-regulation of protein assembly can lead to diseases [5] such as Alzheimer’s. This has inspired researchers to focus on native [6,7] as well as artificial systems [8–10] to understand and re-create these protein assemblies and elucidate the assembly mechanisms. In particular, the artificial protein nanostructures emerged as novel materials for (bio)nanotechnology, enabling the generation of bioactive hydrogels and nanometer-scale electronics [11,12].

In the artificial protein assemblies, native protein-ligand interactions are often selected as a driving force for the formation of higher order protein nanostructures [13–17], due to their accessibility and the ease of adding multiplicity. The use of diverse synthetic elements allows us to broaden the scope of possible shapes and stabilities [18–20], but the formation of protein nanostructures is still driven by the protein-ligand interaction. Supramolecular polymers offer an alternative driving force for assembly, the non-covalent self-assembly of amphiphilic building blocks in water, and have therefore emerged as attractive architectures for protein assembly [21–23]. The non-covalent organization provides entry to responsive materials, facile “supramolecular synthesis” of these materials and incorporating both multiple and different ligands through simple mixing of the supramolecular building blocks. These characteristics make supramolecular polymers an excellent platform for constructing multivalent protein assemblies [24,25].

**Figure 1:**
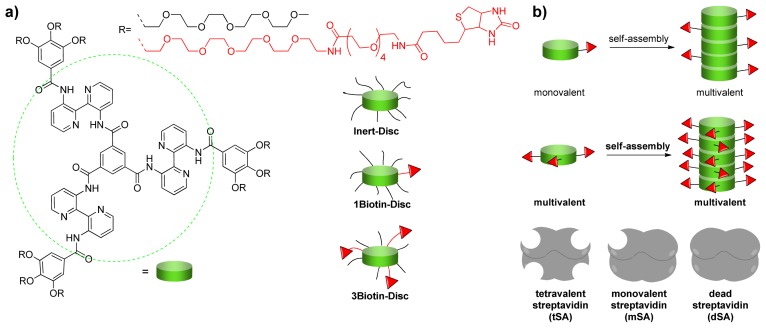
(**a**) Library of non- and biotinylated discotics; (**b**) Self-assembling multivalency of the discotics and the streptavidin mutants used in this study.

The supramolecular polymers used herein are *C_3_*-symmetrical amphiphilic discotics, which self-assemble into auto-fluorescent columnar stacks with a large Stokes shift [26] at dilute micromolar concentrations in water [27]. The monomers consist of an extended aromatic core of three 2,2′-bipyridine-3,3′-diamine molecules linked to a central benzene-1,3,5-tricarbonyl unit (Figure 1a). This hydrophobic core is shielded by nine biocompatible and water soluble poly(ethylene)glycol (PEG) chains, which allows self-assembly in water and prevents unspecific interactions with biological matter. For biological applications, ligands can be introduced at the periphery of the scaffold [22,28]. The functionalization of one discotic with three biotin ligands resulted in a trivalent supramolecular building block, which assembled several streptavidin molecules along the supramolecular polymer [22]. Here, the monovalent variant of this discotic is introduced as an intrinsically non-multivalent building block, which only upon self-assembly generates multivalent polymers. The use of discotics as a platform for protein assembly requires the presence of multiple binding sites or attachment points. Therefore, it is of fundamental interest to understand the self-assembly of intrinsically monovalent discotics into columnar wires and the resulting supramolecular multivalent ligand display. The assembly of monovalent and multivalent streptavidins along this supramolecular polymer is studied to evaluate the characteristics of the self-assembling multivalency [29,30] of discotics.

## 2. Results and Discussion

### 2.1. Design

Previously, it was shown that functionalization of **3NH_2_-Disc** with three biotins (**3Biotin-Disc**) enabled the display of streptavidin, a biotin-binding protein and of an anti-biotin antibody along the supramolecular stack [22]. The binding was confirmed using Förster resonance energy transfer (FRET) from the auto-fluorescent supramolecular polymer to the dye labeled biotin binding protein or antibody. The so-called **Inert-Disc**, which is decorated with PEG chains only, showed no unspecific interactions with the proteins. FRET between differently labeled streptavidins, indicated that upon binding of multiple streptavidins to the supramolecular platform, interactions between the differently labeled streptavidins could be induced. Streptavidin is a tetrameric protein of 52.8 kDa which interacts strongly and specifically with biotin (Kd = 4 × 10^−14^ M) [31]. The tetrameric binding, however, can cause cross-links between several supramolecular wires. To investigate the protein-assembly while minimizing multivalent binding and crosslinking on the one hand a monovalent **1Biotin-Disc** discotic (Figure 1a) was synthesized (see experimental section). On the other hand, next to the tetravalent streptavidin, monovalent streptavidin as well as a non-biotin binding streptavidin mutant [32,33] were used to investigate the assembly of multiple proteins using monovalent self-assembling building blocks and the effects of cross-linking (Figure 1b).

### 2.2. FRET Titrations

**Figure 2:**
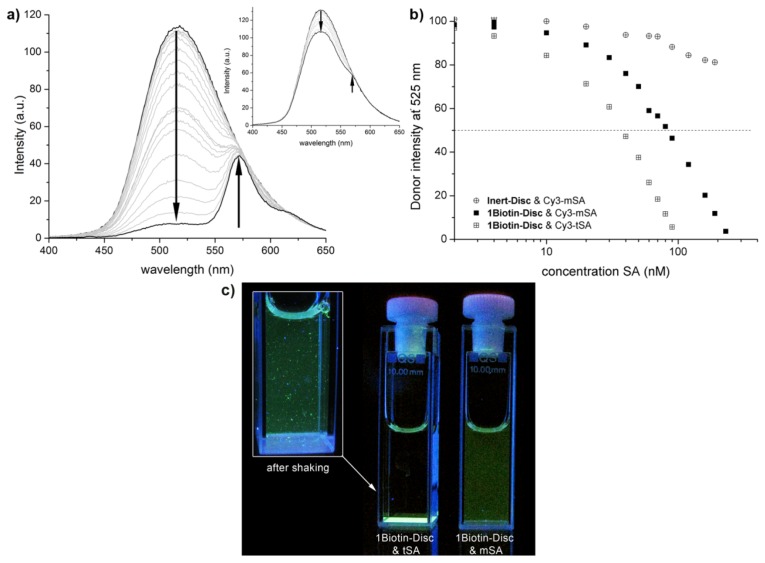
(**a**) Emission spectra of the titration of monovalent Cy3-labeled streptavidin (Cy3-mSA, 0 nM–270 nM) to **1Biotin-Disc** (1 μM). Inset: Emission spectra of the titration of monovalent Cy3-labeled streptavidin (0 nM–270 nM) to **Inert-Disc** (1 μM); (**b**) Normalized decrease in disc intensity at 525 nm upon addition of Cy3-mSA or Cy3-tSA to **1Biotin-Disc** and **Inert-Disc** (both 1 μM); Dashed line represents the intensity, where half of the donor intensity is quenched; (**c**) Photography of cuvettes under UV-light three days after Förster resonance energy transfer (FRET) titration.

Förster resonance energy transfer (FRET) is a non-radiative energy transfer between two fluorescent molecules. The fact, that the efficiency of energy transfer highly depends on the distance between the donor and acceptor molecule [34] led to an extensive use of this technique as a so-called “molecular ruler” to study protein-protein interactions [35–37]. Here, the dye Cy3 was selected as an acceptor, due to a spectral overlap of its absorbance spectrum with the emission spectrum of the discotics, a requirement for energy transfer. Subsequently, the protein assembly of Cy3-labeled monovalent and tetravalent streptavidin (Cy3-mSA and Cy3-tSA) on supramolecular wires of **1Biotin-Disc** was investigated using FRET (Figure 2). The binding of streptavidin to biotin decorated supramolecular wires should lead to close proximity of the dye-labeled protein to the auto-fluorescent discotic scaffold and enable energy transfer from the discotic to the Cy3, resulting in an increase in acceptor signal (575 nm) and a simultaneous decrease in donor signal (525 nm).

Indeed, upon addition of Cy3-labeled monovalent and tetravalent streptavidin to **1Biotin-Disc** an increase in acceptor signal (575 nm) and a simultaneous decrease in donor signal (525 nm) was observed (Figure 2a). No energy transfer was detected between the Cy3-labeled streptavidin and the non-biotinylated **Inert-Disc**, underlining that the observed FRET signal is selectively induced through the binding of Cy3-labeled streptavidins to the appending biotins on the supramolecular scaffold (Figure 2a, inset) [38]. As expected, less of the tetravalent streptavidin was required to fully quench the emission of the donor compared to the monovalent streptavidin, as the Cy3-tSA might lead to more efficient energy transfer through cross-linking between the wires. Plotting the decrease in donor intensity against the concentration of added streptavidin reveals that only 0.04 equivalents (40 nM) of tetravalent streptavidin is sufficed to quench half of the donor fluorescence of the **1Biotin-Disc** (Figure 2b). This indicates that 12 discotics are quenched by one Cy3-tSA at this concentration (donor: acceptor ratio is 25 to 1 and half of donor is quenched). This is twice as much as the 0.02 equivalent which were reported for the quenching of the trivalent **3Biotin-Disc** with tetravalent streptavidin [22]; possibly through binding of several streptavidins to one **3Biotin-Disc** and more efficient cross-linking. In the case of the fully monovalent system (monovalent streptavidin and **1Biotin-Disc**), only 0.09 equivalents (90 nM) of Cy3-mSA suffice to quench half of the donor fluorescence. This implies that at this concentration the fluorescence of approximately 5 discotics is quenched by one Cy3-mSA acceptor fluorophore (donor: acceptor ratio is 11 to 1 and half of donor is quenched). This shows that a multivalent protein-platform is generated through the self-assembly of intrinsically monovalent **1Biotin-Discs**, since for the theoretical 1 to 1 binding of the monovalent protein to the monovalent discotic in the non self-assembled state 0.5 instead of 0.09 equivalents would be required to achieve the same quenching effect.

Overtime the cross-linking of supramolecular polymers through tetravalent streptavidin was visible in the solution (Figure 2c). In contrast the titration solution of the monovalent system of mSA and **1Biotin-Disc** remained homogenously fluorescent without the formation of visible aggregates, even days after titration. When tetravalent streptavidin was used, large aggregates were formed overtime which sedimented on the bottom of the cuvette. Upon shaking, large fluorescent protein cross-linked supramolecular fibers were observed.

### 2.3. Semi-Native SDS-PAGE

**Figure 3:**
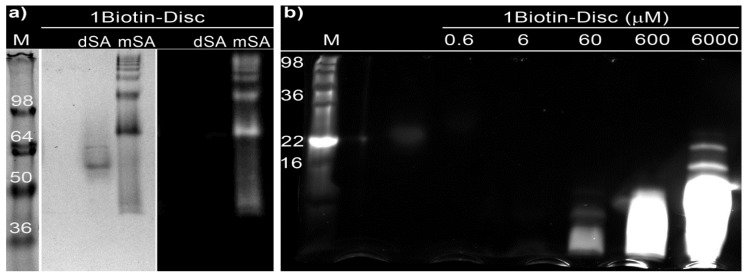
(**a**) Semi-native sodium dodecyl sulfate (SDS)-gel (8%) stained with Coomassie blue (**left**) and under UV-illumination (**right**, λ_ex_ = 350 nm) before staining. Concentration of **1Biotin-Disc** is 20 μM, of dSA and mSA 0.4 mg/mL; (**b**) Semi-native SDS-gel (15%) under UV-illumination of different concentrations of the **1Biotin-Disc** only.

Additionally, the ability of self-assembled **1Biotin-Disc** to bind monovalent streptavidin(s) was investigated using non-reducing sodium dodecyl sulfate polyacrylamide gel electrophoresis (SDS-PAGE) [39]. In this widely used technique, proteins are separated according to their electrophoretic mobility. The electrophoretic mobility is a function of the length and of the charge of the protein. Even distribution of charge per mass unit is guaranteed through binding of SDS, enabling the separation of proteins according to their size. Denaturation of streptavidin through addition of a reducing agent (β-mercaptoethanol) and heating to 95 °C (a standard procedure for SDS-PAGE) would result in the dissociation of the subunits and the simultaneous dissociation of bound biotin. Consequently a non-reducing approach was chosen to visualize the binding of monovalent streptavidin to the supramolecular polymer consisting of **1Biotin-Disc**. Prior to electrophoresis the **1Biotin-Disc** was incubated with monovalent streptavidin and as a control with dead streptavidin (dSA), which consists only of non-biotin binding subunits. The semi-native SDS gel was imaged under UV-illumination (λ_ex_ = 350 nm) and subsequently the proteins were stained with Coomassie blue (Figure 3a).

The **1Biotin-Disc** itself, with a molecular weight of 3.5 kDa, was not detectable on this gel (Figure 3a). Dead streptavidin incubated with **1Biotin-Disc** showed one non-fluorescent band corresponding to the tetravalent streptavidin of 55 kDa without any **1Biotin-Disc** bound. In the case of the mixture of monovalent streptavidin with **1Biotin-Disc** several higher mass protein bands were observed, which at the same time featured fluorescence when excited at 350 nm, resulting from the bound **1Biotin-Disc**. This can be explained through the binding of several monovalent streptavidins to a supramolecular wire consisting of several self-assembling **1Biotin-Discs**, since the strong auto-fluorescence is only observed in the self-assembled state. This observation suggests that the protein-decorated supramolecular wires which are formed through self-assembly of discotics are at least partially stable under the SDS-PAGE conditions. The occurrence of up to seven discrete protein bands indicates as well the assembly of multiple discotics into longer stacks, which enable the binding of at least seven monovalent streptavidins. The formation of discrete protein bands is in contrast to biotinylated polymers [40], which when bound to labeled streptavidin result in a smear on the gel. This indicated that the formed supramolecular polymers are much smaller than the size of streptavidin, which through their larger size are the main size determining factor. The formation of supramolecular polymers was first confirmed with Dynamic Light Scattering (DLS) (see experimental section). To further elucidate the size of supramolecular polymers, the electrophoretic mobility of the **1Biotin-Disc** was investigated using a separating gel with a higher acrylamide percentage, to test if the supramolecular polymers themselves and their size-distribution can be visualized. Only at concentrations higher than 60 μM, the fluorescence of the discotics was visible at the bottom of the gel, *i.e.*, below 16 kDa (Figure 3b). With increasing concentration and gel loading, the band of the discotics became larger, indicating the presence of larger supramolecular polymers and at the highest concentration (6 mM) even distinct bands of 16 and 22 kDa were visible. Monomer concentration thus can have an influence on the size of supramolecular polymers, as also reported elsewhere [41].

**Figure 4:**
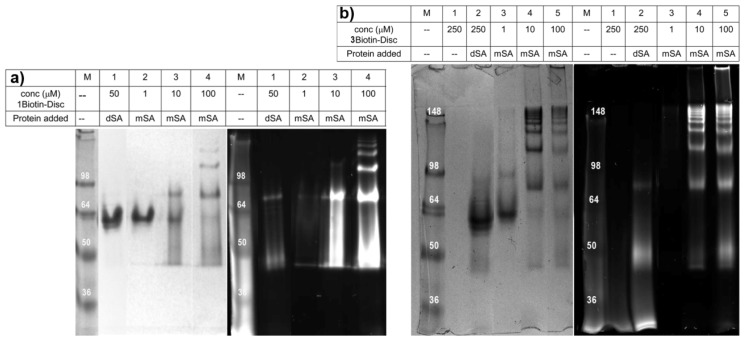
(**a**) Semi-native SDS-gel (8%) of different concentrations of **1Biotin-Disc** and mSA (5 μM) or dSA (5 μM) stained with Coomassie blue (**left**) and under UV-illumination (**right**) before staining; (**b**) Semi-native SDS-gel (8%) of different concentrations of **3Biotin-Disc** and mSA (5 μM) or dSA (5 μM) stained with Coomassie blue (**left**) and under UV-illumination (**right**) before staining.

To elucidate the effect of discotic monomer concentration, semi-native SDS-gels were recorded using a constant concentration of the protein mSA (5 μM) and with increasing concentrations of **1Biotin-** or **3Biotin-Disc** ranging from 1 to 100 μM (Figure 4). The dead streptavidin (dSA) showed even at a 10- or 50-fold excess of biotinylated discotics no unspecific binding, since only a single and non-fluorescent protein band was observed. In contrast, with the increasing concentration of biotinylated discotics the appearance of several fluorescent protein bands was observed in the case of the monovalent streptavidin. The 1 μM concentration of both, **1Biotin-** and **3Biotin-Discs**, seems to be too low to form supramolecular polymers displaying several proteins under these SDS-PAGE conditions. Only at the concentration of 10 μM and higher was the appearance of higher molecular weight bands observed. In the case of **1Biotin-Disc**, at 10 μM concentration, only one additional band is observed, indicating mainly the binding of two mSA to the supramolecular polymers of **1Biotin-Disc**. For the appearance of up to seven additional bands, 100 μM of **1Biotin-Disc** was required (Figure 4a). With the **3Biotin-Disc**, which offers two additional streptavidin binding sites per molecule, over seven additional bands are already observed at 10 μM. At higher concentrations of **3Biotin-Disc,** such large assemblies are formed that they remain located at the top of the gel (Figure 4b).

**Figure 5:**
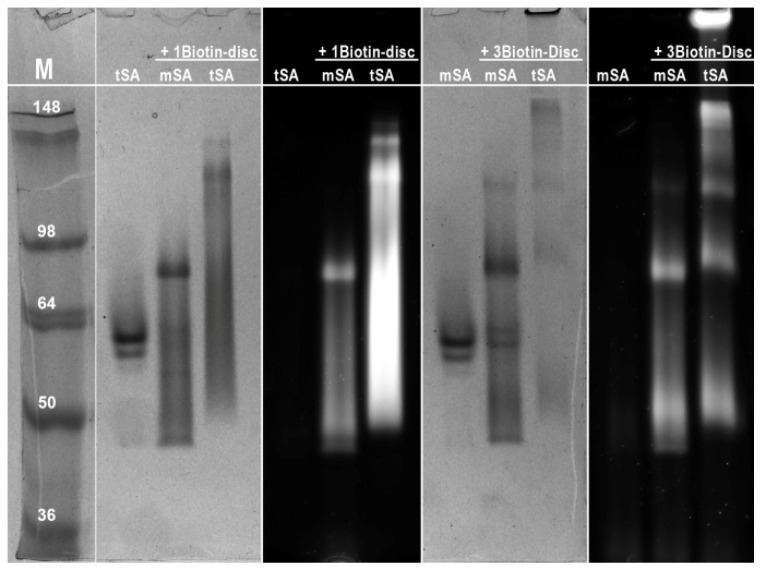
Semi-native SDS-gel (8%) of **1Biotin-Disc** and **3Biotin-Disc** (both 100 μM) with mSA (5 μM) and tSA (5 μM) stained with Coomassie blue and under UV-illumination before staining.

Next, the formed protein-assemblies of different discotics with monovalent and tetravalent streptavidin were compared at a disc concentration of 100 μM (Figure 5). At this concentration, in all cases, the binding of streptavidin to discotics led to higher molecular weight bands. The use of tetravalent streptavidin always resulted in the generation of larger constructs, and when both multivalent systems were combined, the cross-linked supramolecular polymers were too large to enter the cross-linked gel, as already observed in solution (Figure 2c).

## 3. Experimental Section

### 3.1. Materials and Methods

NHS-PEG_4_-biotin was purchased from Pierce, Thermo Scientific. All other solvents and chemicals were purchased from Sigma-Aldrich (Zwijndrecht, The Netherlands) and used as received. Water was demineralized prior to use. Dichloromethane (DCM) (HPLC grade) was degassed with argon and purified by passage through activated alumina solvent column prior to use. Analytical thin layer chromatography (TLC) was carried out using Merck pre-coated silica gel or aluminium oxide plates (60F-254) using ultraviolet light irradiation at 254 or 365 nm. Manual size-exclusion chromatography was performed on BIO RAD BioBeads S-X1 (200–400 mesh) in a long glass column (1.2 m) at atmospheric pressure and a flow rate less than 1 mL/min in dimethylformamide (DMF). Matrix assisted laser desorption/ionisation time of flight mass spectra (MALDI-TOF-MS) were measured on a PerSeptive Biosystems Voyager-DE PRO spectrometer with a Biospectrometry workstation using 2-[(2E)-3-(4-tert-butylphenyl)-2-methylprop-2-enylidene]malononitrile (DCTB) and α-Cyano-4-hydroxycinnamic acid (CHCA) as matrix material and THF as solvent. *M*/*z* values are given in g/mol. ^1^H and ^13^C NMR spectra were recorded using a Varian Mercury Vx 400 MHz (100 MHz for ^13^C) NMR spectrometer at 298 K. Chemical shifts are given in parts per million (ppm) and the spectra are calibrated to residual solvent signals of CDCl_3_ (7.26 ppm (^1^H) and 77 ppm (^13^C)). UV-vis spectra were measured on a Jasco V-650 spectrophometer. Fluorescence spectra were recorded on a Varian Cary Eclipse fluorescence spectrophotometer equipped with a Perkin–Elmer PTP-1 Peltier temperature control system. All UV-vis and fluorescence measurements were performed in quartz cuvettes of 10 mm light path (Hellma, Müllheim, Germany) and 2 mL minimal volume at 20 °C. **3Biotin-Disc**[22], **1NH_2_-Disc**[23] and **Inert-Disc**[27] were synthesized according to literature.

### 3.2. Synthesis of 1Biotin-Disc

A solution of NHS-PEG_4_-biotin (4.2 mg, 7.2 μmol) in dry dichloromethane (0.2 mL) was added dropwise to a solution of **1NH_2_-Disc** (7.9 mg, 2.4 μmol) and triethyl amine (10 μL, 72 μmol) in dry dichloromethane (0.5 mL) and the reaction was continued overnight. Full conversion was observed with TLC (silica, dichloromethane with 10% methanol, stained with Seebach reagent, *R*_f_ = 0.27). After concentrating the reaction mixture *in vacuo*, the **1Biotin-Disc** was purified via size-exclusion chromatography (BioBeads SX-1 in DMF, Bio-Rad, Veenendaal, The Netherlands) yielding pure compound (5 mg, 1.3 μmol, 54%). ^1^H–NMR (CDCl_3_): δ = 15.53 (s, 3H), 14.49 (s, 3H), 9.60 (d, *J* = 7.5 Hz, 3H), 9.39 (d, *J* = 8.6 Hz, 3H), 9.29 (s, 3H), 9.06 (d, *J* = 4.4 Hz, 3H), 8.52 (d, *J* = 3.5 Hz, 3H), 7.57 (dd, *J* = 8.4, 4.6 Hz, 6H), 7.36 (s, 6H), 6.84 (s, 1H), 6.61 (s, 1H), 5.56 (s, 1H), 5.12 (s, 1H), 4.49 (dd, *J* = 6.9, 5.5 Hz, 1H), 4.33 (m, 1H), 4.27 (dd, *J* = 10.7, 5.6 Hz, 18H), 3.90 (t, *J* = 4.7 Hz, 12H), 3.83 (t, *J* = 4.7 Hz, 6H), 3.76–3.51 (m, 174H), 3.43 (m, 5H), 3.37 (s, 6H), 3.35 (s, 18H), 3.20 – 3.09 (m, 1H), 2.91 (dd, J = 12.8, 5.1 Hz, 1H), 2.72 (d, *J* = 12.8 Hz, 1H), 2.48 (t, *J* = 6.1 Hz, 2H), 2.21 (t, *J* = 6.9 Hz, 2H), 2.03 (s, 1H), 1.88 (s, 6H), 1.80–1.28 (m, 6H). ^13^C NMR (CDCl_3_): δ = 173.16, 171.51, 165.98, 164.26, 152.88, 142.52, 142.41, 141.69, 140.89, 137.68, 137.58, 136.30, 130.71, 130.09, 129.61, 124.85, 124.36, 108.18, 72.66, 72.07, 72.05, 70.98, 70.81, 70.77, 70.71, 70.66, 70.64, 69.88, 69.55, 67.47, 61.90, 60.21, 59.16, 55.44, 40.65, 39.31, 37.01, 35.82, 28.19, 25.36. MALDI-ToF MS: *m/z* calcd (C_181_H_278_N_16_O_67_S) 3782.28; found 3804.81 [M + Na]^+^, 3820.80 [M + K]^+^. Longtime storage results in partial oxidation of the biotin, as observed with MALDI-ToF (Bruker, Bremen, Germany), which doesn’t diminish the binding affinity to streptavidin [42]. MS: *m*/*z* calcd. (C_181_H_278_N_16_O_68_S) 3795.85; found 3796.5 [M]^+^.

### 3.3. DLS of 1Biotin- and 3Biotin-Disc

**Figure 6:**
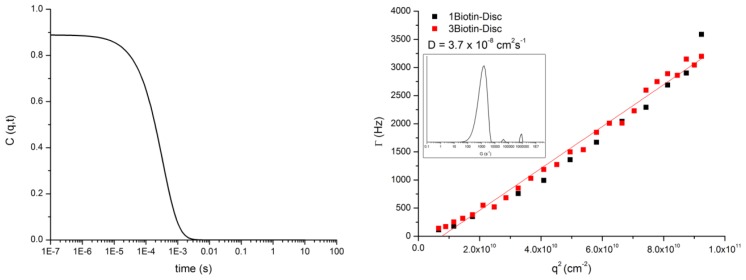
**Left**: Plot of the autocorrelation function of the **1Biotin-Disc** at the 90° angle. **Right**: Plot of q2 *vs.* gamma of **1Biotin-** and **3Biotin-Disc**. From the q2 *vs.* gamma plots, the diffusion coefficient was extracted. The inset shows the distribution of species at the 90° angle for the **1Biotin-Disc**.

Dynamic light scattering experiments (DLS) (Figure 6) were performed on an ALVCGS-3 Compact Goniometer (ALV, Langen, Germany), in the angular range of 25 to 151 degrees. The incident beam was produced by a HeNe laser (ALV, Langen, Germany) operating at 632 nm. The intensity signal was sent to an ALV5000 digital correlator (ALV, Langen, Germany), using a typical acquisition time of 100 s for each angle.

### 3.4. Expression and Labeling of Streptavidin

Plasmids for the “dead”- and “alive”-subunit were obtained from Alice. Y. Ting (Massachusetts Institute of Technology) and monovalent streptavidin was expressed following a published protocol [32]. The concentration of monovalent and tetravalent streptavidin (purchased from Sigma, Zwijndrecht, The Netherlands) in PBS was adjusted to 3 mg/mL (determined by NanoDrop, Thermo Scientific, Wilmington, DE, USA); mass: 55 kDa, extinction coefficient: 200.000 M^−1^ cm^−1^). 0.723 mg Cy3-NHS (purchased from Lumiprobe, Hannover, Germany) was dissolved in 10 μL DMF. The pH of the mSA and tSA in PBS was set to 8.5 and 5 μL of Cy3-NHS in DMF was added to both reaction mixtures. The reaction was performed overnight at room temperature and in the dark. The excess of dye was removed through dialysis.

### 3.5. FRET Titrations

**Figure 7:**
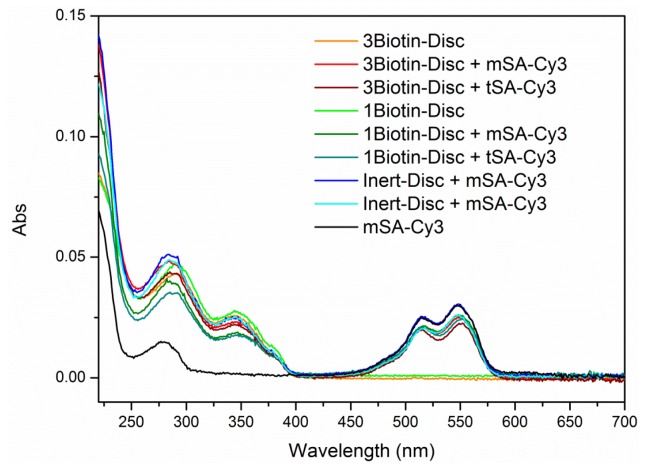
Absorption spectra after addition of 70 nM of corresponding protein during FRET titrations.

Cy3 labeled monovalent or tetravalent streptavidin (4 μM in PBS) was titrated to **1Biotin-Disc** or **3Biotin-Disc** or **Inert-Disc** (each 1 μM in PBS) in serial concentrations from 2 nM to 310 nM at 20 °C. After each addition the solution in the cuvette was intermixed by turning the closed cuvette several times upside-down. Fluorescence- (λ_ex_ = 340 nm, λ_em_ = 400–650 nm) spectra were measured after each titration step. Additionally, UV absorption spectra were measured after addition of 70 nM of corresponding protein (see Figure 7) to confirm correct concentrations.

### 3.6. Semi-Native SDS-PAGE

Semi-native SDS-PAGE electrophoresis was performed on a Mini-PROTEAN 3 electrophoresis system (Biorad, Hercules, CA, USA). The gel consisted of an 8% separating gel and a 5% stacking gel. For the **1Biotin-Disc** only gel in Figure 3b a gel consisting of 15% separating gel and 5% stacking gel was used. The running buffer contained 25 mM Tris, 250 mM Glycine, and 0.1% (*w/v*) SDS in H_2_O. Monovalent and dead streptavidin were incubated for about 16 hours at room temperature with different concentrations of **1Biotin-Disc** and **3Biotin-Disc**. No β-mercaptoethanol was added to the samples and the samples were not heated up to 95 °C to avoid protein denaturation and the disassembly of the tetrameric streptavidin into single subunits. Sample buffer (100 mM Tris-HCl, 20% (*v/v*) glycerol, 4% (*w/v*) SDS, 0.2% (*w/v*) bromophenol blue, pH 6.8 in Millipore H_2_O) was added (1:3) to the samples and electrophoresis was run at room temperature at 80 V for 30 min and then at 140 V for 60 min. The protein bands were stained with Coomassie Brilliant Blue. To visualize the discotics, the gels were photographed under UV-light prior to Coomassie Brilliant Blue staining. SeeBlue^®^ Plus2 pre-stained standard (purchased from Invitrogen, Bleiswijk, The Netherlands) was used as a marker.

## 4. Conclusions

A novel monovalent supramolecular discotic building block was generated, which self-assembled in water into multivalent columnar supramolecular polymers. Studies performed on supramolecular polymers formed by this monovalent discotic, decorated with a single biotin, and analogous trivalent discotics with streptavidin constructs of different valency, revealed the nature of the self-assembling multivalency of these systems with regard to the assembly of proteins. The possibility to display several monovalent streptavidins using supramolecular polymers based on the monovalent **1Biotin-Disc** was confirmed with FRET measurements and semi-native SDS-PAGE. The self-assembly of the monovalent biotinylated discotics induces the assembly of several proteins along the supramolecular polymer, without inducing cross-links between polymers. Tuning the concentration of the discotics influences the length of the resulting polymers and thus dictates the amount of assembled proteins. The portfolio of both monovalent and trivalent supramolecular building blocks in combination with monovalent and multivalent streptavidins thus allows the tuning of the resulting supramolecular polymer–protein hybrids regarding size and protein density. Such supramolecular protein platforms, with their ease of synthesis and tuning of molecular properties, are thus excellent entries for future applications of supramolecular materials in controlling protein aggregation and function.
